# Tetrathiomolybdate inhibits mitochondrial complex IV and mediates degradation of hypoxia-inducible factor-1α in cancer cells

**DOI:** 10.1038/srep14296

**Published:** 2015-10-15

**Authors:** Kyu Kwang Kim, Sarah Abelman, Naohiro Yano, Jennifer R. Ribeiro, Rakesh K. Singh, Marla Tipping, Richard G. Moore

**Affiliations:** 1 Molecular Therapeutics Laboratory, Program in Women’s Oncology, Departments of Obstetrics and Gynecology, Women and Infants Hospital, Alpert Medical School of Brown University, Providence, RI, USA; 2 Department of Biology, Providence College, Providence, RI, USA

## Abstract

Hypoxia-inducible factor-1α (HIF-1α) is a transcription factor that triggers adaptive responses upon low oxygen conditions and plays a crucial role in cancer metabolism and therapy resistance. Tetrathiomolybdate (TM), a therapy option for copper overload disorder, has also been shown to be capable of limiting tumor angiogenesis, although its underlying mechanism remains unclear. Using ovarian and endometrial cancer cell lines, we observed that TM downregulates HIF-1α protein levels and HIF-transcriptional targets involved in tumor angiogenesis and glycolysis, but did not affect HIF-1α protein synthesis. TM-mediated HIF-1α downregulation was suppressed when HIF-prolyl hydroxylase activity was pharmacologically inhibited using deferoxamine or dimethyloxaloylglycine, and also when the oxygen-dependent degradation domains of HIF-1α, which are responsible for the interaction with HIF-prolyl hydroxylase, were deleted. These findings suggest that TM causes HIF-1α downregulation in a HIF-prolyl hydroxylase-dependent manner. Our studies showed that TM inhibits the activity of the copper-dependent mitochondrial complex IV and reduces mitochondrial respiration, thereby possibly increasing oxygen availability, which is crucial for HIF-prolyl hydroxylase activity. Pimonidazole staining also showed that TM elevates oxygen tension in hypoxic cells. Our studies provide mechanistic evidence for TM-mediated HIF-1α regulation and suggest its therapeutic potential as a method of blocking angiogenesis in ovarian and endometrial tumors.

Nutrients and oxygen delivered through the vascular system are critical for tumor growth. Without vascular support, tumors cannot grow beyond 1–2 mm^3^. Therefore, angiogenesis, the development of new blood vessels, is a critical process in tumor growth and spread. One way that this process is mediated in cancerous tissues is through regulation by the protein hypoxia-inducible factor-1α (HIF-1α). HIF-1α is known to trigger adaptive responses during low oxygen conditions, thereby transcriptionally activating many genes involved in many aspects of cancer metabolism including angiogenesis, invasion, metastasis, glycolysis, tumor survival, and proliferation^1^. In addition, overexpression of HIF-1α has been considered a poor prognostic factor in several different types of malignancies^2^. Thus, HIF-1α is considered a promising target for cancer treatment.

HIF-1 is a heterodimeric protein consisting of HIF-1α and HIF-1β subunits. Under hypoxic or low-oxygen conditions, HIF-1α is stabilized and localized into the nucleus where it heterodimerizes with HIF-1β. The HIF-1α/HIF-1β complex recognizes the HIF-responsive elements (HREs) of its target genes and binds to coactivators, such as CBP/p300, to mediate gene expression^3^. Oxygen affects HIF-1 activity through proline and asparagine hydroxylation. The hydroxylation of two proline residues (Pro^402^ and Pro^564^) within the oxygen-dependent degradation domain (ODD) of HIF-1α by HIF-prolyl hydroxylases, also termed prolyl hydroxylase domains (PHDs), is required for recognition by the von Hippel-Lindau (VHL) protein. VHL interaction with HIF-1α leads to HIF-1α protein degradation through the ubiquitin-proteasome pathway. In addition, HIF-1α asparagine hydroxylation (Asn^803^) by the protein factor inhibiting HIF-1 (FIH-1) prevents CBP/p300 binding to HIF-1α, thereby blocking transcriptional activations of target genes. These processes by PHDs and FIH-1 require oxygen and 2-oxoglutarate as well as other cofactors such as Fe^2+^ and ascorbate^3^,^4^. Therefore, tumors under hypoxic conditions stabilize HIF-1 and promote its transcriptional activities.

Studies have demonstrated the association between copper and angiogenesis. In tumor xenograft models, levels of ceruloplasmin, a serum copper marker, were found to correlate with tumor development and metastatic spread^5^. Several other *in vitro* and *in vivo* studies support the theory that copper plays a role in angiogenesis^6^,^7^. In humans it has been proposed that cancer may elevate serum copper levels^8^. In one study, patients with breast cancer showed higher copper levels than those with benign diseases and those in the control group^9^. Several proof-of-concept model studies have supported the angiogenic role of copper. Copper was shown to be required for HIF-1α activation^10^, induce VEGF expression in cells, and promote wound repair in mice^11^. With this in mind, a therapeutic modality to deplete copper levels in tumors may have a potent anti-angiogenic effect on tumor cells and be a novel approach for the treatment of cancer.

Several clinical copper chelators have been developed in the search for the treatments of copper overload disorders such as Wilson’s disease. Among the copper chelators, tetrathiomolybdate (TM or ATN-224), trientine and D-penicillamine, have all shown anti-cancer therapeutic potentials, suggesting that copper deprivation is a promising option for cancer treatment. Toxicity from copper deficiency by TM is known to be minimal or can be reversed promptly^12^. In a recent phase II study in patients with breast cancer, oral daily doses of TM were administered for up to 2 years^13^. TM, as monotherapy or in combination with other treatment modalities, can be useful for the treatment of cancer. Several cell and animal model studies by us^14^,^15^ and others^16^,^17^ have found that TM exerts potent anti-cancer effects and improves therapeutic responses to other anti-cancer drugs. Specifically in terms of the effects of TM on tumor angiogenesis, it has been reported that TM is capable of reducing microvessel density in different types of tumors^16^,^18^. TM inhibited metastasis induced by tail vein tumor injection^19^ and has also been shown to inhibit release of several angiogenic factors in cells and to suppress angiogenesis in a rat aortic ring assay^16^. Rats treated with TM demonstrate decreased HIF-1α expression^20^. However, the molecular mechanism of HIF-1α regulation by TM treatment remains to be determined. This study explores the underlying mechanism by which TM mediates degradation of HIF-1α and identifies a therapeutic advantage for the use of TM in targeting chemo-resistant cancer cells.

## Results

### Treatment with TM downregulates HIF-1α protein levels and suppresses HIF-target genes

**Figure 1:**
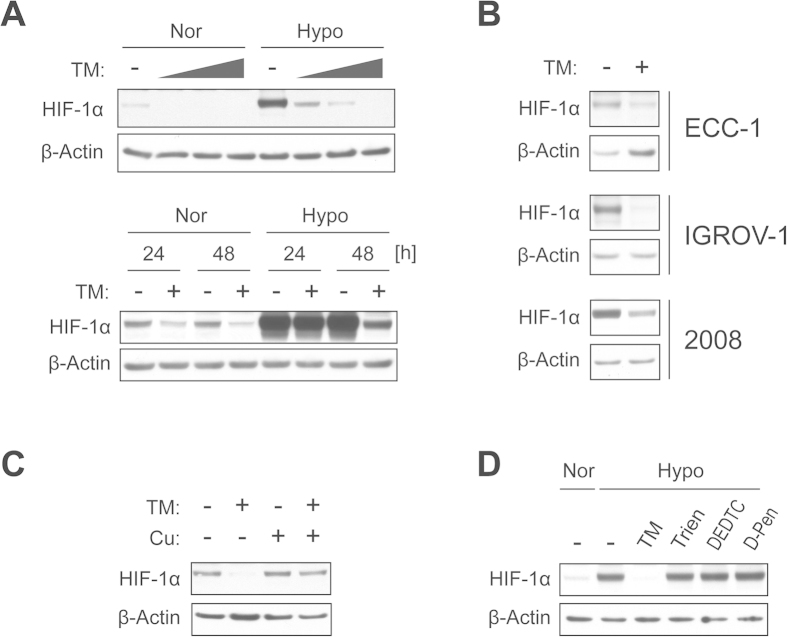
TM mediates downregulation of HIF-1α protein levels. (**A**) ECC-1 cells were treated with various concentrations of TM (0, 3.3, 10, 30 μM) for 48 h (top) or treated with 10 μM of TM for different durations (24, 48 h) (bottom) under normoxic or hypoxic (2% O_2_) conditions. (**B**) ECC-1, IGROV-1 and 2008 cells were untreated or treated with TM (30 μM) for 24 h. (**C**) ECC-1 cells were treated with or without TM (30 μM) in the presence or absence of excess Cu^2+^ (90 μM) for 48 h. (**D**) ECC-1 cells were untreated or treated with TM (30 μM) and other copper chelators such as trientine (Trien), diethyldithiocarbamate (DEDTC) and D-penicillamine (D-Pen) at a 3-fold higher concentration (90  μM) under hypoxic conditions for 48 h. Cells exposed to normoxia were included as reference. After finishing each treatment condition, cell lysates were collected and subjected to immunoblotting to determine HIF-1α protein levels. Expression of β-actin served as a loading control.

**Figure 2:**
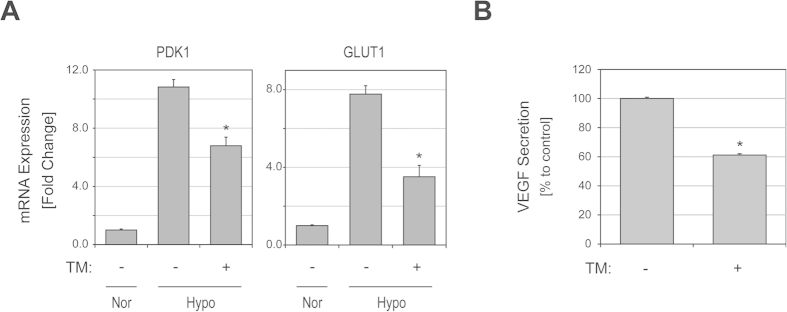
TM treatment suppresses downstream targets of HIF-1α. (**A**) ECC-1 cells were cultured under normoxic or hypoxic (2% O_2_) conditions in the presence or absence of TM (30 μM) for 48 h. mRNA expression of HIF-1α target genes (PDK1 and GLUT1) was analyzed using real-time PCR. β-actin was used as reference for normalization. (*P < 0.05 versus hypoxic control, *N *= 3) (**B**) ECC-1 cells were treated with or without TM (30 μM) under hypoxic (2% O_2_) conditions for 42 h. Growth media was replenished, collected after 6 h and subjected to VEGF analysis. (*P < 0.05 versus control from three independent experiments; Representative data is shown)

To assess whether TM treatment suppresses the HIF signaling pathway in gynecologic cancer cells, we first determined the effects of TM on HIF-1α protein levels using immunoblotting in ECC-1 human endometrial cancer cells. TM mediated a dose- and time-dependent reduction of HIF-1α protein levels (Fig. 1A). HIF-1α levels in ECC-1 cells were clearly decreased after TM treatment for 48 h at a concentration as low as 3.3 μM (lower concentrations were not evaluated), under both normoxic and hypoxic (2% O_2_) conditions (Fig. 1A upper). Levels of HIF-1α were markedly decreased within 24 h of TM treatment (10 μM) under normoxic conditions. Under hypoxic conditions, no significant reduction in HIF-1α levels was observed for a 24 h TM incubation; however, after 48 h HIF-1α levels in ECC-1 cells were clearly decreased (Fig. 1A lower). Following these findings of TM effects on levels of HIF-1α in ECC-1 cells, we studied other gynecologic cancer cells and assessed effects of TM treatment on HIF-1α levels. Similar to that seen in ECC-1 cells, TM treatment (30 μM, 24 h) under normoxia effectively reduced the levels of HIF-1α in IGROV-1 and 2008 human ovarian cancer cells (Fig. 1B), suggesting that the effects of TM on levels of HIF-1α are not cell-specific events. Next, in order to evaluate if copper deprivation contributed to the alteration in HIF-1α levels following TM treatment, ECC-1 cells were treated with TM (30 μM, 48 h) in the absence or presence of excess amounts of copper (90 μM), and immunoblotting was carried out against HIF-1α. The TM-mediated decrease in HIF-1α levels was clearly restored by the presence of copper (Fig. 1C), indicating that copper deprivation by TM caused the decrease in HIF-1α levels in ECC-1 cells. Interestingly, other clinical and experimental copper chelating drugs such as trientine, diethyldithiocarbamate, and D-penicillamine did not result in a decrease in HIF-1α levels, even at a three-fold higher concentration than what was used for TM treatment (Fig. 1D). In order to see if TM also affects transcriptional activations of HIF-1α target genes, we conducted real-time PCR to measure mRNA levels after TM treatment. ECC-1 cells exposed to hypoxia (2% O_2_) markedly increased expression of glycolytic genes PDK1 and GLUT1 (Fig. 2A). These HIF-1α-regulated genes in hypoxic ECC-1 cells were clearly suppressed by TM treatment. In addition, the secretion of angiogenic factor VEGF was also decreased in the presence of TM (Fig. 2B).

### Treatment with TM causes HIF-1α downregulation in a PHD-dependent manner

**Figure 3:**
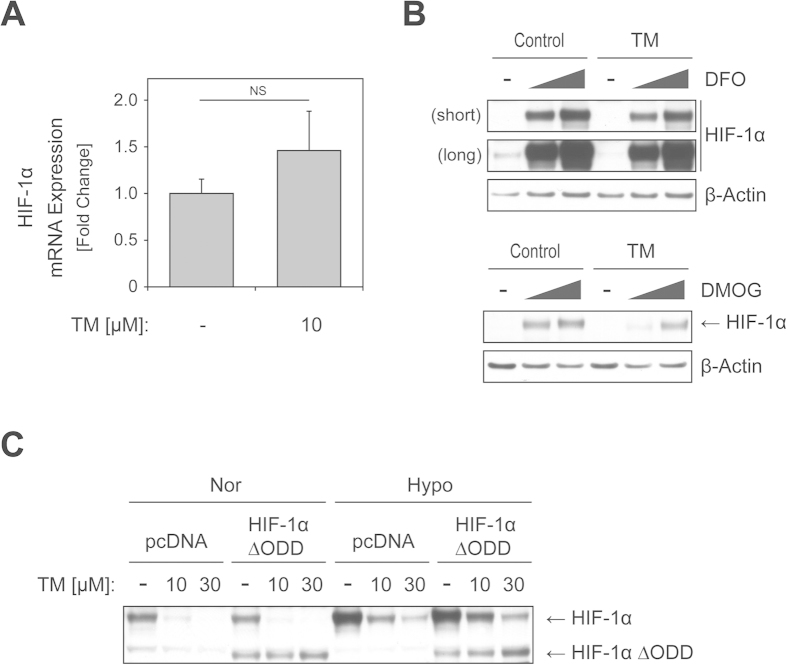
TM affects HIF-1α in a PHD-dependent manner. (**A**) ECC-1 cells were treated with or without TM (0, 10 μM) for 8 h. mRNA expression of HIF-1α was analyzed using real-time PCR. β-actin was used as reference for normalization. (NS = Not significant; P > 0.1 versus control, *N *= 3) (**B**) ECC-1 cells were untreated or treated with TM (30 μM) in the presence or absence of PHD inhibitors at a range of concentrations for 48 h; deferoxamine (DFO: 0, 17, 50 μM) and dimethyloxaloylglycine (DMOG: 0, 1, 2 mM). HIF-1α protein levels were determined by immunoblotting. β-actin expression served as a loading control. (**C**) ECC-1 cells were transfected with control vector or a construct expressing an HIF-1α mutant (HIF-1α ∆ODD) lacking its interaction with PHD. After 24 h, cells were exposed to TM treatment (0, 10, 30 μM) under normoxic or hypoxic (2% O_2_) conditions for an additional 48 h. Cell lysates were collected and subjected to immunoblotting with HIF-1α antibody.

To explore the mechanism by which TM treatment regulates HIF-1α protein levels, we first sought to determine if TM affects the mRNA level of HIF-1α in ECC-1 cells. ECC-1 cells were incubated with or without TM, and HIF-1α mRNA expression was analyzed using real-time PCR. TM (10 μM, 8 h) did not reduce HIF-1α mRNA levels (Fig. 3A), which is consistent with previous findings^20^. Next, we examined if TM affects HIF-1α through a PHD-dependent mechanism as PHD plays a crucial role in HIF-1α degradation^3^. Fe^2+^ and 2-oxoglutarate are essential for PHD activity and therefore, we blocked PHD activity using either an iron chelator, deferoxamine (DFO), or a 2-oxoglutarate analog, dimethyloxaloylglycine (DMOG), and then assessed their effects on HIF-1α levels with or without TM treatment. The ability of TM to decrease HIF-1α protein levels was partially reduced when cells were co-treated with DFO or DMOG (Fig. 3B), suggesting that TM-mediated downregulation of HIF-1α protein possibly occurs through mechanisms that involve PHD. In order to provide further support for this hypothesis, we employed ECC-1 cells transiently expressing a HIF-1α ∆ODD mutant, in which the oxygen-dependent degradation domain of HIF-1α is deleted, preventing its interactions with PHD and thereby rendering HIF-1α resistant to PHD/VHL-mediated degradation. As shown in Fig. 3C, TM clearly downregulated levels of wild-type HIF-1α, but did not alter levels of HIF-1α ∆ODD, strongly supporting the idea that TM targets HIF-1α through a PHD-dependent mechanism.

### TM inhibits mitochondrial complex IV and alters cellular oxygen tension

**Figure 4:**
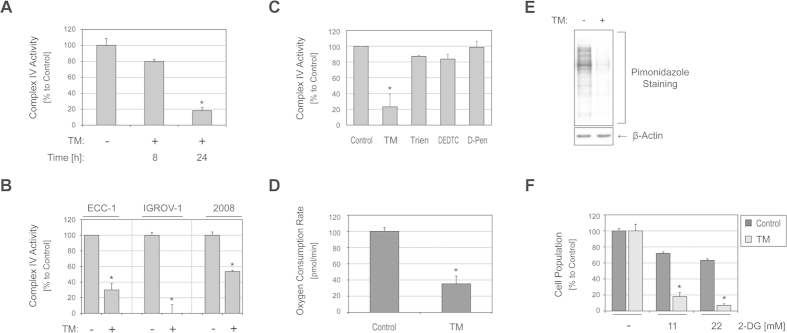
TM treatment suppresses mitochondrial respiration through inhibition of the mitochondrial respiratory chain complex IV. (**A**) ECC-1 cells were untreated or treated with TM (30 μM) for the indicated durations (8, 24 h). (**B**) ECC-1, IGROV-1 and 2008 cells were treated with or without TM (30 μM) for 24 h. (**C**) ECC-1 cells were untreated or treated with TM (30 μM) and other copper chelators; Trien, DEDTC and D-Pen at a 3-fold higher concentration for 24 h. After treatment, cells were harvested and subjected to complex IV activity assay ((**A–C)**: *P < 0.05 versus untreated control from at least two independent experiments; Representative data is shown, error bars: SD). (**D**) ECC-1 cells were untreated or treated with TM (30 μM), after which oxygen consumption rates were monitored. The data represent the relative values from control and TM treated cells after 12 h incubation (*P < 0.05 versus control, *N *= 3, error bars: SEM). (**E**) ECC-1 cells were incubated with or without TM (30 μM) under hypoxic (2% O_2_) conditions for 48 h. Relative oxygen tension in cells was evaluated by immunoblotting with an antibody specific to pimonidazole protein adducts. (**F**) ECC-1 cells were incubated with or without TM (30 μM) for 24 h after which the cells at each condition were exposed to 2-deoxyglucose (0, 11, 22 mM) for an additional 24 h. Cell population was determined using SRB assay as indicated in “Materials”. Data represents the mean values relative to each control (*P < 0.05 versus 2-DG only, *N *= 6, error bars: SD).

PHD activity is regulated in an oxygen-dependent manner. Mitochondrial respiration consumes the majority of cellular oxygen and thus affects oxygen tension in cells. Alterations in mitochondrial respiration are known to affect PHD activity and HIF-1α protein levels^21^. Therefore, we sought to determine if TM causes a change in mitochondrial respiration and oxygen consumption in cells. Complex IV (or cytochrome c oxidase) of the mitochondrial respiratory chain is a copper-dependent enzyme. Thus, TM treatment could alter the activity of complex IV. We found that treatment with TM decreased the activity of complex IV in ECC-1 cells (Fig. 4A). We then considered other cell lines in which TM treatment was found to reduce HIF-1α protein levels. We treated IGROV-1 and 2008 cells with or without TM and analyzed their complex IV activities. Similar to what we observed in ECC-1 cells, complex IV activities in both cell lines were markedly decreased by TM treatment (Fig. 4B). Next, we treated ECC-1 cells with a set of different copper chelators (Fig. 1D) to assess their effects on complex IV activity. Our findings suggested that TM is far more effective at suppressing complex IV activity in ECC-1 cells compared to other copper chelators tested even at a concentration three-fold higher than what was used for TM treatment (Fig. 4C). This observation also correlates well with our observation that the other copper chelators did not downregulate HIF-1α levels (Fig. 1D).

Blockage of the mitochondrial respiration chain has been proposed to increase cellular oxygen availability toward PHD thereby destabilizing HIF-1α protein even under hypoxic conditions^22^. To this end, we first tested if TM treatment alters oxygen consumption by ECC-1 cells. Cells were incubated with or without TM, and rates of oxygen consumption were monitored and calculated. As shown in Fig. 4D, oxygen consumption was lower in TM treated ECC-1 cells than in untreated cells. We speculated that the lowered oxygen consumption may increase available cellular oxygen to PHD. To test this hypothesis, we stained for pimonidazole, an oxygen-sensitive probe that has widely been used as a hypoxic marker in *in vitro* and *in vivo* models^23^. Pimonidazole is reductively activated to produce immunoreactive protein adducts. Oxygen inhibits the activation of pimonidazole. ECC-1 cells were exposed to hypoxic conditions either in the absence or presence of TM treatment. Following this treatment, cell lysates were collected and subjected to immunoblotting using an antibody against pimonidazole adducts. As shown in Fig. 4E, ECC-1 cells exposed to hypoxia clearly showed the formation of pimonidazole adducts, which were evidently decreased when cells were co-incubated with TM, suggesting that TM increases oxygen concentrations in hypoxic ECC-1 cells. Taken together, our findings suggested that treatment with TM causes HIF-1α protein degradation through inhibition of mitochondrial respiration and subsequent alterations in cellular oxygen tension. Of note, other respiratory inhibitors such as oligomycin (ATP synthase) or sodium azide (complex IV) similarly decreased HIF-1α protein levels in ECC-1 cells (Suppl. Fig. 1), implicating mitochondrial respiration in HIF-1α regulation in ECC-1 cells.

The production of mitochondrial reactive oxygen species (ROS) is known to cause HIF-1α stabilization. To test if ROS accounts for HIF-1α stability in ECC-1 cells and if TM blocks ROS production, we treated ECC-1 cells with H_2_O_2_ at a concentration (200 μM) found sufficient to increase intracellular ROS levels by flow cytometry after staining with a ROS dye carboxy-H_2_DCFDA. Interestingly, we found that H_2_O_2_ treatment did not upregulate HIF-1α protein levels in ECC-1 cells (Suppl. Fig. 2A). Furthermore, TM treatment did not suppress cellular ROS levels, but rather enhanced them (Suppl. Fig. 2B). Next we investigated if TM-induced inhibition of mitochondrial respiration leads to changes in energy pathway utilization. When mitochondrial respiration is limited, cancer cells may rely more on the glycolytic pathway for energy production and thus become sensitive toward therapeutic inhibition of glycolysis. To test this hypothesis, ECC-1 cells were treated with TM and 2-deoxyglucose, a non-metabolizable glucose analog that blocks glycolysis, as a single agent or in combination, and cell populations in each condition were determined. Our data showed that TM can enhance inhibitory effects of 2-deoxyglucose (Fig. 4F), suggesting its therapeutic potential when combined with other therapies including those that target the glycolytic pathway.

### TM downregulates HIF-1α independent of AKT signaling pathway

**Figure 5:**
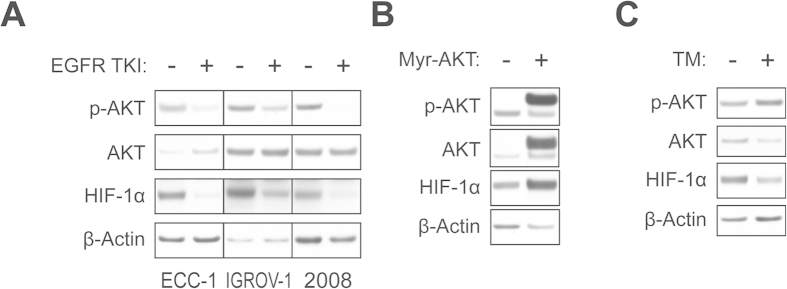
Inhibition of PI3K/AKT pathway decreases HIF-1α protein levels and TM mediates AKT-independent HIF-1α downregulation. (**A**) ECC-1, IGROV-1 and 2008 cells were treated with or without EGFR tyrosine kinase inhibitor (TKI) AG1478 (5 μM, 18 h) in serum free medium. Cell lysates were collected and subjected to immunoblotting to determine p-AKT, AKT, and HIF-1α protein levels. Expression of β-actin served as a loading control. (**B**,**C**) 2008 cells were transfected with a myristoylated AKT (Myr-AKT) or empty vector for 48 h in 1% FBS medium (**B**) or were treated with or without TM (30 μM) for 24 h incubation under the same culture condition as indicated in (**B**) (**C**). Cell lysates were collected and subjected to immunoblotting against the proteins as indicated.

Numerous anti-HIF-1α therapeutic strategies have been based on their ability to block HIF-1α protein synthesis^1^. However, genetic alterations such as PTEN, a negative regulator of AKT, and PIK3CA, a catalytic subunit of phosphatidylinositol-3-kinase (PI3K), are often found in cancers including endometrial and ovarian cancers. Increased PI3K-AKT-mTOR signaling resulting from these mutations can increase HIF-1α synthesis^1^ and may render cancer cells resistant to anti-HIF-1α strategies, such as those targeting receptor tyrosine kinases^4^,^24^. To gain insight into the role of AKT signaling in HIF-1α regulation in the cell lines used in our study, we incubated ECC-1, IGROV-1, and 2008 cells with AG1478, an EGFR tyrosine kinase inhibitor, and analyzed AKT activation and HIF-1α protein levels. Our data revealed that all three cell lines tested show a decrease in AKT phosphorylation upon EGFR inhibition, which was also accompanied by a reduction in HIF-1α levels (Fig. 5A). To further understand the role of AKT signaling on HIF-1α, we transiently expressed myristoylated AKT and measured levels of HIF-1α protein. Our preliminary study suggested that transfection efficiency of myristoylated AKT was low in ECC-1 cells (data not shown), but was robust in 2008 cells. We found that expression of myristoylated AKT markedly increases HIF-1α protein levels in 2008 cells (Fig. 5B), suggesting that AKT activation in 2008 cells contributes to HIF-1α synthesis. In order to see if AKT signaling is also required for TM-induced downregulation of HIF-1α, we treated 2008 cells with or without TM and evaluated HIF-1α levels and AKT activation. Our data suggested that TM-mediated HIF-1α downregulation was AKT-independent since TM treatment did not decrease AKT phosphorylation in 2008 cells while concurrently suppressing HIF-1α protein levels (Fig. 5C).

## Discussion

Angiogenesis plays a pivotal role in tumor growth and metastasis. Numerous studies have provided evidence that TM treatment has anti-cancer and anti-angiogenic potentials^14^,^15^,^16^,^17^,^18^,^19^. In this study we sought to explore the underlying mechanisms through which TM inhibits tumor angiogenesis, with special attention to its ability to suppress HIF-1α protein expression. We chose ovarian and endometrial cancer cells as our experimental models, as anti-angiogenic therapies are validated for ovarian cancer treatment. For instance, bevacizumab (an anti-VEGF monoclonal antibody) plus chemotherapy is known to improve clinical outcomes in patients with ovarian cancer^25^. In addition, levels of VEGF expression in endometrial cancer were found to be higher than those of benign and atypical hyperplasia tissue^26^, and increased VEGF levels were associated with poor outcomes in patients with a subtype of endometrial cancer^27^. This suggests that strategies to target angiogenesis could be a promising therapeutic option for the treatment of endometrial cancer.

In the current study, we showed that TM inhibits HIF-1α protein accumulation and impairs the activation of its transcriptional targets relating to glucose metabolism and angiogenesis. In search of mechanistic actions of TM in HIF-1α regulation, we found that TM treatment in ECC-1 cells did not appear to affect HIF-1α protein synthesis. However, TM blocked HIF-1α protein accumulation during hypoxia and also partially compromised HIF-1α levels elevated by PHD inhibitors such as DFO and DMOG. Furthermore, protein expression of the HIF-1α ∆ODD mutant that lacked its interactions with PHD, was not affected by TM, while TM clearly downregulated protein expression of wild-type HIF-1α, indicating that PHD plays a crucial role in TM-mediated HIF-1α degradation. To further define these findings, we determined if TM affects mitochondria and PHD-guided oxygen sensing due to the link between mitochondrial respiration, regulation of PHD activity, and HIF-1α stabilization during hypoxia. An elegant study by Hagen *et al.*^22^ has proposed that the inhibition of mitochondrial respiration using nitric oxide, an endogenous complex IV inhibitor, or other respiratory inhibitors shifts intracellular oxygen availability toward PHD, thereby destabilizing HIF-1α protein. In this study, we observed that TM treatment clearly inhibits the activity of complex IV in the ovarian and endometrial cancer cells used in our study, which also correlated with a reduction in HIF-1α levels. In addition, TM markedly lowered cellular oxygen consumption, which possibly led to an increase in oxygen availability for PHD. Finally, pimonidazole staining indicated that oxygen concentrations in cells elevate after TM treatment. Together, our data suggested that TM mediates HIF-1α degradation through mitochondrial inhibition.

It is possible that alterations in cellular ROS levels may affect HIF-1α stability during TM treatment. The seminal study by Chandel *et al.*^28^ has proposed that generation of mitochondrial ROS plays a crucial role in cellular response to hypoxia. ROS may deplete cellular Fe^2+^, which is required for PHD activity, through its oxidation into Fe^3+^, thereby inactivating PHD protein and leading to the stabilization of HIF-1α^29^. To this end, we treated cells with H_2_O_2_ at concentrations sufficient to elevate cellular ROS levels, yet failed to stabilize HIF-1α. We cannot rule out the possibility that TM also affects HIF-1 transcriptional activation because copper deprivation is known to reduce HIF-1α binding to HRE and to inhibit formation of HIF-1 transcriptional complex with p300^10^.

Targeting HIF-1 has gained attention as an attractive target for cancer therapy over the past several years. A number of different approaches have been proposed to inhibit HIF-1 by targeting different steps of HIF-1 regulation, including HIF-1α mRNA expression, HIF-1α protein synthesis, maintenance of HIF-1α protein stability, HIF-1α/HIF-1β dimerization, HIF-1 DNA binding, and HIF-1 transactivation. An overview of those inhibitors is provided in a recent review^4^. Many of these small molecule inhibitors are known to target HIF-1α protein synthesis by blocking mTOR signaling or receptor tyrosine kinases such as EGFR, HER2, and BCR/ABL, thereby blocking downstream mTOR indirectly. However, oncogenic alterations of PTEN and PIK3CA are often found in ovarian and endometrial cancers. For instance, PTEN mutation is the most common genetic lesion, found in up to 83% of endometrioid endometrial cancers^30^. PIK3CA mutation was also reported in 36% of endometrial carcinomas^31^. These mutations may lead to an increase in mTOR activity and thus HIF-1α protein synthesis, which may render cancer cells resistant to anti-HIF-1α therapies that affect upstream regulators of HIF-1α synthesis, such as those targeting receptor tyrosine kinases. In this paper, we evaluated a proof-of-concept experimental cell model to simulate such mutations by enhancing AKT activity. An increase in AKT signaling indeed elevated HIF-1α levels. Conversely, TM mediated HIF-1α degradation through a mechanism independent of AKT deactivation, suggesting that TM may provide a therapeutic advantage to bypass the intervening mutations in those therapy-resistant tumors.

It has been known for decades that many tumors can produce energy through glycolysis, even in the presence of an adequate oxygen supply, known as the Warburg effect. However, oxidative phosphorylation was shown to contribute to about 65–95% of total ATP production in many cancer cells, in particular up to 95% in ovarian and uterine cancer^32^. Inhibition of the mitochondrial respiratory chain has been proposed as a mechanistic target of several anti-cancer compounds^33^,^34^. BAY 87–2243, a potent HIF-1 inhibitor in phase I clinical trial, is known to inhibit mitochondrial complex I^35^. Likewise, the biguanide class drug metformin that is one of the most widely used for patients with Type 2 diabetes is known to target complex I^33^. Accumulating evidence suggests that metformin may have therapeutic potential for cancer treatment. Many retrospective clinical studies found that metformin decreases cancer risk in diabetic patients^33^. Metformin regulates the AMPK/mTOR pathway, possibly through complex I inhibition, interferes with energy metabolism and protein synthesis, and inhibits cancer growth^33^. Although metformin inhibits HIF-1 activation^36^, this drug is also known to inhibit AKT signaling^37^. To this end, treatment with TM may provide several different benefits over metformin treatment. In this study, TM-mediated HIF-1α inhibition is independent of AKT-deactivation. TM inhibits complex IV, which is the rate-determining enzyme of the mitochondrial respiration chain and thus a key site for cellular energy production^38^. Also, cancer cells may require more copper than normal cells^39^,^40^. A recent study by Ishida *et al.*^40^ demonstrated that mice fed with elevated amounts of copper display an increase in tumor growth, which was impaired by copper deprivation with TM. Importantly, they also found that tumors in TM-treated mice showed a decrease in complex IV activity, supporting the translational potential of our findings.

It is interesting that TM was more effective in inhibiting complex IV activity in ECC-1 cells than other copper chelators. The stability constants for trientine and DEDTC toward Cu^2+^ are known to be higher (20.4 and 14.9 respectively) than that of TM (8.0), while that of D-pen is comparable (7.1)^41^. TM, unlike D-pen, is considered a membrane-permeable chelator^42^, possibly more effective in targeting cellular cuproenzymes directly. However, DEDTC, which is also cell-permeable, did not inhibit complex IV in our study^43^. It is known that DEDTC binds to other metals such as zinc. To this end, future studies will be needed to determine selectivity toward different metals and/or metalloenzymes and their stabilities as well as relative permeability of each chelator. It would be intriguing to see if TM is more effective in targeting cellular cuproenzymes directly. We cannot rule out the possibility that long-term copper deprivation with other chelators could affect complex IV activity as evidenced in neuroblastoma cell culture using trientine^44^. In conclusion, due to our findings and those of the aforementioned studies, further studies involving xenograft models of ovarian and endometrial cancers are warranted to evaluate *in vivo* efficacy of TM and to investigate the interplay between complex IV and HIF-1α following TM therapy.

## Materials

### Cell lines, cell culture and reagents

ECC-1 (RPMI-1640) and IGROV-1 (DMEM) cell lines were purchased from the American Type Culture Collection. 2008 (RPMI-1640) was kindly provided by Dr. François X. Claret (University of Texas M. D. Anderson Cancer Center). Cells were cultured in media (specified in parentheses), supplemented with 10% fetal calf serum, penicillin (100 units/mL), and streptomycin (100 μg/mL) and maintained at 37 °C with 5% CO_2_ in a humidified incubator. Hypoxia (2% O_2_) was generated using the ProOx controller (model C21, BioSpherix) with the help of N_2_ and CO_2_ infusion gases, in a sealed and humidified chamber and maintained at 5% CO_2_ and 37 °C. Reagents and antibodies were purchased as follows: All the chemicals were purchased from Sigma Aldrich unless otherwise stated; carboxy-H_2_DCFDA (Life technologies); AG1478, p-AKT (Ser473) and AKT (Cell Signaling Technology); β-actin (Santa Cruz Biotechnology); HIF-1α (Novus Biologicals); Pimonidazole and its MAb1 (NPI). Human VEGF Quantikine ELISA Kit was purchased from R&D Systems and used for detection of VEGF secretion following manufacturer’s recommendations.

### Quantitative real-time PCR

Cells were lysed and total RNA was isolated using Trizol reagent (Invitrogen). Reverse transcription was performed using SuperScript III First-Strand Synthesis System (Invitrogen), both following the manufacturer’s recommendations. Quantitative real-time PCR was carried out using Taqman Gene Expression Master Mix and the following Taqman Gene Expression Assay (Applied Biosystem) containing FAM-labeled probes: GLUT1, Hs00892681_m1; PDK1, Hs01561850_m1; HIF-1α, Hs00153153_m1. An assay targeting β-actin with a VIC-labeled probe (Hs01060665_g1) was used as reference for normalization. Relative gene expression was calculated using the 2^−∆∆CT^ method. Reactions were carried out using the following thermal cycling conditions: 50 °C for 2 minutes and 95 °C for 10 minutes, then 40 cycles of 95 °C for 15 seconds and 60 °C for 1 minute.

### Measurement of complex IV activity

Mitochondrial Complex IV (Human) Activity Assay Kit (Millipore; AAMT004) was used to measure the activity of Complex IV. The assay was conducted following manufacturer’s recommendations. Complex IV activity was determined as a reduction in absorbance at 550 nm by calculating the difference between the initial and end value.

### Measurement of cell population and ROS

ECC-1 cells were treated with or without TM, 2-deoxyglucose, H_2_O_2_ or their combinations at the concentrations and durations as indicated. For cell population studies, we employed sulforhodamine B (SRB) assay. In brief, after the drug treatment was finished, cells were fixed in 10% cold trichloroacetic acid, washed with water, stained with SRB, following the protocol as previously detailed^14^. In order to measure cellular ROS levels, we stained cells with a ROS indicator carboxy-H_2_DCFDA dye (Invitrogen). Following the drug treatment cells were washed with PBS and incubated with carboxy-H_2_DCFDA (15 μM) at 37 °C for 20 min. Cells were harvested by trypsinization, resuspended in PBS and analyzed using a FACS Canto flow cytometer with Diva software (BD Biosciences).

### Determination of oxygen consumption rate

Oxygen consumption rate was measured using the Seahorse XF-96e Flux Analyzer (Seahorse Biosciences, North Billerica, MA). ECC-1 cells were plated at a density of 10,000 cells/well in a Seahorse 96-well cell culture plate and allowed to adhere to the culture plate surface. Cells were washed with Seahorse Assay media, consisting of modified unbuffered DMEM (Seahorse Biosciences, North Billerica, MA, catalog # 102352-000) containing 10 mM pyruvate and 10 mM glucose. Cells were equilibrated with this assay media at 37 °C and degassed for 30 minutes. Oxygen consumption rate was measured using 3-minute measurement periods over 24 hours. Relative oxygen consumption rates after 12 hours of incubation were graphed for control and TM treated ECC-1 cells.

### Immunoblotting

Cells were transfected with pcDNA3 or pcDNA3-HIF-1α/∆ODD (a kind gift of Dr. Eric Huang; the University of Utah)^45^ or with a myristoylated AKT expression construct (Myr-AKT; Millipore) or empty vector (pUSEamp). Transient transfection was carried out with lipofectamine 2000 or 3000 (Invitrogen), following the manufacturer’s recommendations. Cells were washed with cold-PBS twice and lysed in Cell Lysis Buffer (Cell Signaling Technology) supplemented with 1 mM PMSF. Protein concentration was determined using Bio-Rad DC Protein Assay Kit (BioRad). Protein samples were resolved by NuPAGE Gel system using a 4–12% Tris-Bis gradient gel and MES SDS running buffer (Invitrogen), transferred onto a PVDF membrane (BioRad), blocked with 5% nonfat dry milk (BioRad) in TBS-Tween 20 (0.1%) buffer, and probed against their respective antibodies as indicated in individual experiments. Blots were visualized with ECL Prime Western Blotting Detection Reagents (GE Healthcare). Stripping of blots was performed using OneMinute Advance Western Blot Stripping buffer (GM Biosciences).

### Statistical Analysis

Data was analyzed using t-test. P < 0.05 was considered statistically significant.

## Additional Information

**How to cite this article**: Kim, K. K. *et al.* Tetrathiomolybdate inhibits mitochondrial complex IV and mediates degradation of hypoxia-inducible factor-1a in cancer cells. *Sci. Rep.* **5**, 14296; doi: 10.1038/srep14296 (2015).

## Supplementary Material

Supplementary Information

## References

[b1] Semenza G. L. Targeting HIF-1 for cancer therapy. Nat. Rev. Cancer 3, 721–732 (2003). 13130303 10.1038/nrc1187

[b2] Semenza G. L. HIF-1 mediates metabolic responses to intratumoral hypoxia and oncogenic mutations. J. Clin. Invest 123, 3664–3671 (2013). 23999440 10.1172/JCI67230 PMC3754249

[b3] Ke Q. & Costa M. Hypoxia-inducible factor-1 (HIF-1). Mol. Pharmacol. 70, 1469–1480 (2006). 16887934 10.1124/mol.106.027029

[b4] Semenza G. L. Hypoxia-inducible factors: mediators of cancer progression and targets for cancer therapy. Trends Pharmacol. Sci. 33, 207–214 (2012). 22398146 10.1016/j.tips.2012.01.005 PMC3437546

[b5] Ungar-Waron H., Gluckman A., Spira E., Waron M. & Trainin Z. Ceruloplasmin as a marker of neoplastic activity in rabbits bearing the VX-2 carcinoma. Cancer Res. 38, 1296–1299 (1978). 639063

[b6] Alessandri G., Raju K. & Gullino P. M. Angiogenesis *in vivo* and selective mobilization of capillary endothelium *in vitro* by heparin-copper complex. Microcirc. Endothelium Lymphatics 1, 329–346 (1984). 6546149

[b7] Raju K. S., Alessandri G., Ziche M. & Gullino P. M. Ceruloplasmin, copper ions, and angiogenesis. J. Natl. Cancer Inst. 69, 1183–1188 (1982). 6182332

[b8] Coates R. J., Weiss N. S., Daling J. R., Rettmer R. L. & Warnick G. R. Cancer risk in relation to serum copper levels. Cancer Res. 49, 4353–4356 (1989). 2743325

[b9] Gupta S. K., Shukla V. K., Vaidya M. P., Roy S. K. & Gupta S. Serum trace elements and Cu/Zn ratio in breast cancer patients. J. Surg. Oncol. 46, 178–181 (1991). 2011029 10.1002/jso.2930460311

[b10] Feng W., Ye F., Xue W., Zhou Z. & Kang Y. J. Copper regulation of hypoxia-inducible factor-1 activity. Mol. Pharmacol. 75, 174–182 (2009). 18842833 10.1124/mol.108.051516 PMC2685058

[b11] Sen C. K. *et al.* Copper-induced vascular endothelial growth factor expression and wound healing. Am. J. Physiol Heart Circ. Physiol 282, H1821–H1827 (2002). 11959648 10.1152/ajpheart.01015.2001

[b12] Brewer G. J. The promise of copper lowering therapy with tetrathiomolybdate in the cure of cancer and in the treatment of inflammatory disease. J. Trace Elem. Med. Biol. 28, 372–378 (2014). 25194954 10.1016/j.jtemb.2014.07.015

[b13] Jain S. *et al.* Tetrathiomolybdate-associated copper depletion decreases circulating endothelial progenitor cells in women with breast cancer at high risk of relapse. Ann. Oncol. 24, 1491–1498 (2013). 23406736 10.1093/annonc/mds654 PMC3707432

[b14] Kim K. K. *et al.* Tetrathiomolybdate induces doxorubicin sensitivity in resistant tumor cell lines. Gynecol. Oncol. 122, 183–189 (2011). 21529906 10.1016/j.ygyno.2011.03.035

[b15] Kim K. K., Lange T. S., Singh R. K., Brard L. & Moore R. G. Tetrathiomolybdate sensitizes ovarian cancer cells to anticancer drugs doxorubicin, fenretinide, 5-fluorouracil and mitomycin C. BMC. Cancer 12, 147 (2012). 22502731 10.1186/1471-2407-12-147 PMC3353246

[b16] Pan Q. *et al.* Copper deficiency induced by tetrathiomolybdate suppresses tumor growth and angiogenesis. Cancer Res. 62, 4854–4859 (2002). 12208730

[b17] Pan Q., Bao L. W., Kleer C. G., Brewer G. J. & Merajver S. D. Antiangiogenic tetrathiomolybdate enhances the efficacy of doxorubicin against breast carcinoma. Mol. Cancer Ther. 2, 617–622 (2003). 12883034

[b18] Cox C. *et al.* The role of copper suppression as an antiangiogenic strategy in head and neck squamous cell carcinoma. Laryngoscope 111, 696–701 (2001). 11359142 10.1097/00005537-200104000-00024

[b19] Kumar P. *et al.* Tetrathiomolybdate inhibits head and neck cancer metastasis by decreasing tumor cell motility, invasiveness and by promoting tumor cell anoikis. Mol. Cancer 9, 206 (2010). 20682068 10.1186/1476-4598-9-206 PMC2922193

[b20] Mizuno S. *et al.* Copper deficiency induced emphysema is associated with focal adhesion kinase inactivation. PLoS. One. 7, e30678 (2012). 22276220 10.1371/journal.pone.0030678 PMC3262830

[b21] Taylor C. T. Mitochondria and cellular oxygen sensing in the HIF pathway. Biochem. J. 409, 19–26 (2008). 18062771 10.1042/BJ20071249

[b22] Hagen T., Taylor C. T., Lam F. & Moncada S. Redistribution of intracellular oxygen in hypoxia by nitric oxide: effect on HIF1alpha. Science 302, 1975–1978 (2003). 14671307 10.1126/science.1088805

[b23] Varghese A. J., Gulyas S. & Mohindra J. K. Hypoxia-dependent reduction of 1-(2-nitro-1-imidazolyl)-3-methoxy-2-propanol by Chinese hamster ovary cells and KHT tumor cells *in vitro* and *in vivo*. Cancer Res. 36, 3761–3765 (1976). 986241

[b24] Li X. *et al.* Requirement of hypoxia-inducible factor-1alpha down-regulation in mediating the antitumor activity of the anti-epidermal growth factor receptor monoclonal antibody cetuximab. Mol. Cancer Ther. 7, 1207–1217 (2008). 18483308 10.1158/1535-7163.MCT-07-2187

[b25] Shaw D., Clamp A. & Jayson G. C. Angiogenesis as a target for the treatment of ovarian cancer. Curr. Opin. Oncol. 25, 558–565 (2013). 23942301 10.1097/CCO.0b013e328363e0da

[b26] Holland C. M., Day K., Evans A. & Smith S. K. Expression of the VEGF and angiopoietin genes in endometrial atypical hyperplasia and endometrial cancer. Br. J. Cancer 89, 891–898 (2003). 12942123 10.1038/sj.bjc.6601194 PMC2394472

[b27] Kamat A. A. *et al.* Clinical and biological significance of vascular endothelial growth factor in endometrial cancer. Clin. Cancer Res. 13, 7487–7495 (2007). 18094433 10.1158/1078-0432.CCR-07-1017

[b28] Chandel N. S. *et al.* Mitochondrial reactive oxygen species trigger hypoxia-induced transcription. Proc. Natl. Acad. Sci. USA 95, 11715–11720 (1998). 9751731 10.1073/pnas.95.20.11715 PMC21706

[b29] Gerald D. *et al.* JunD reduces tumor angiogenesis by protecting cells from oxidative stress. Cell 118, 781–794 (2004). 15369676 10.1016/j.cell.2004.08.025

[b30] Mutter G. L. *et al.* Altered PTEN expression as a diagnostic marker for the earliest endometrial precancers. J. Natl. Cancer Inst. 92, 924–930 (2000). 10841828 10.1093/jnci/92.11.924

[b31] Oda K., Stokoe D., Taketani Y. & McCormick F. High frequency of coexistent mutations of PIK3CA and PTEN genes in endometrial carcinoma. Cancer Res. 65, 10669–10673 (2005). 16322209 10.1158/0008-5472.CAN-05-2620

[b32] Zu X. L. & Guppy M. Cancer metabolism: facts, fantasy, and fiction. Biochem. Biophys. Res. Commun. 313, 459–465 (2004). 14697210 10.1016/j.bbrc.2003.11.136

[b33] Ben S. I., Le Marchand-Brustel Y., Tanti J. F. & Bost F. Metformin in cancer therapy: a new perspective for an old antidiabetic drug? Mol. Cancer Ther. 9, 1092–1099 (2010). 20442309 10.1158/1535-7163.MCT-09-1186

[b34] Xiao D., Powolny A. A. & Singh S. V. Benzyl isothiocyanate targets mitochondrial respiratory chain to trigger reactive oxygen species-dependent apoptosis in human breast cancer cells. J. Biol. Chem. 283, 30151–30163 (2008). 18768478 10.1074/jbc.M802529200 PMC2573064

[b35] Ellinghaus P. *et al.* BAY 87-2243, a highly potent and selective inhibitor of hypoxia-induced gene activation has antitumor activities by inhibition of mitochondrial complex I. Cancer Med . 2, 611–624 (2013). 24403227 10.1002/cam4.112 PMC3892793

[b36] Viollet B. *et al.* Cellular and molecular mechanisms of metformin: an overview. Clin. Sci. (Lond) 122, 253–270 (2012). 22117616 10.1042/CS20110386 PMC3398862

[b37] Zakikhani M., Blouin M. J., Piura E. & Pollak M. N. Metformin and rapamycin have distinct effects on the AKT pathway and proliferation in breast cancer cells. Breast Cancer Res. Treat. 123, 271–279 (2010). 20135346 10.1007/s10549-010-0763-9

[b38] Arnold S. The power of life–cytochrome c oxidase takes center stage in metabolic control, cell signalling and survival. Mitochondrion. 12, 46–56 (2012). 21640202 10.1016/j.mito.2011.05.003

[b39] Farquharson M. J. *et al.* The distribution of trace elements Ca, Fe, Cu and Zn and the determination of copper oxidation state in breast tumour tissue using muSRXRF and muXANES. Phys. Med. Biol. 53, 3023–3037 (2008). 18490810 10.1088/0031-9155/53/11/018

[b40] Ishida S., Andreux P., Poitry-Yamate C., Auwerx J. & Hanahan D. Bioavailable copper modulates oxidative phosphorylation and growth of tumors. Proc. Natl. Acad. Sci. USA 110, 19507–19512 (2013). 24218578 10.1073/pnas.1318431110 PMC3845132

[b41] Ding X., Xie H. & Kang Y. J. The significance of copper chelators in clinical and experimental application. J. Nutr. Biochem. 22, 301–310 (2011). 21109416 10.1016/j.jnutbio.2010.06.010

[b42] White C., Lee J., Kambe T., Fritsche K. & Petris M. J. A role for the ATP7A copper-transporting ATPase in macrophage bactericidal activity. J. Biol. Chem. 284, 33949–33956 (2009). 19808669 10.1074/jbc.M109.070201 PMC2797165

[b43] Tang E. H. *et al.* Calcium and reactive oxygen species increase in endothelial cells in response to releasers of endothelium-derived contracting factor. Br. J. Pharmacol. 151, 15–23 (2007). 17351662 10.1038/sj.bjp.0707190 PMC2012974

[b44] Lombardo M. F., Ciriolo M. R., Rotilio G. & Rossi L. Prolonged copper depletion induces expression of antioxidants and triggers apoptosis in SH-SY5Y neuroblastoma cells. Cell Mol. Life Sci. 60, 1733–1743 (2003). 14513838 10.1007/s00018-003-3153-1 PMC11138813

[b45] Huang L. E., Gu J., Schau M. & Bunn H. F. Regulation of hypoxia-inducible factor 1alpha is mediated by an O2-dependent degradation domain via the ubiquitin-proteasome pathway. Proc. Natl. Acad. Sci. USA 95, 7987–7992 (1998). 9653127 10.1073/pnas.95.14.7987 PMC20916

