# Supplementary material for: Tetrathiomolybdate inhibits mitochondrial complex IV and mediates degradation of hypoxia-inducible factor-1α in cancer cells
Source: Sci Rep. 2015 Oct 15;5:14296. doi: 10.1038/srep14296 (PMC4606568; doi:10.1038/srep14296)

**Tetrathiomolybdate inhibits mitochondrial complex IV and mediates degradation of hypoxia-inducible factor-1α in cancer cells**

Kyu Kwang Kim^1*^, Sarah Abelman^1^, Naohiro Yano^1^, Jennifer R. Ribeiro^1^, Rakesh K. Singh^1^, Marla Tipping^2^, Richard G. Moore^1^

^1^: Molecular Therapeutics Laboratory, Program in Women's Oncology, Departments of Obstetrics and Gynecology, Women and Infants Hospital, Alpert Medical School of Brown University, Providence, RI, USA

^2^: Department of Biology, Providence College, Providence, RI, USA

^*^: Corresponding Author

**
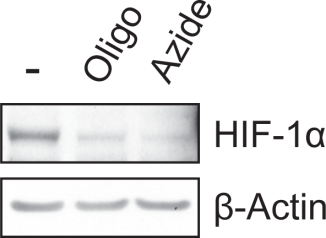
**

**Figure 1. (Suppl.):** ECC-1 cells were untreated or treated with oligomycin (500 nM) and sodium azide (5 mM) for 3 h, after which cell lysates were prepared and subjected to immunoblotting to measure HIF-1α protein levels. β-actin served as a loading control.


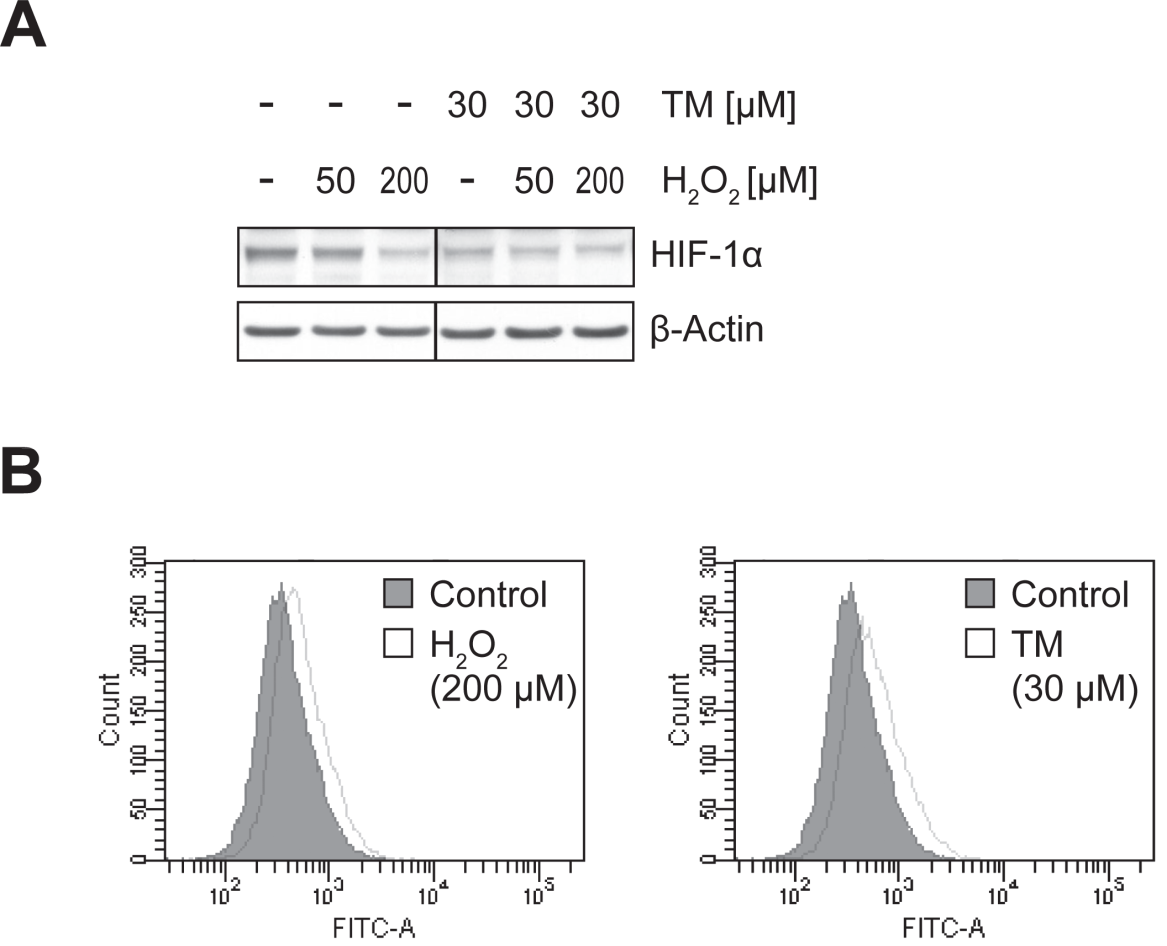


**Figure 2. (Suppl.):** ECC-1 cells were incubated with or without TM (30 µM) for 24 h, after which the cells at each condition were exposed to H_2_O_2_ at the concentrations as indicated for additional 3 h. After the treatment was completed, cell lysates were collected and subjected to immunoblotting against the proteins as indicated (A) or cells were stained with a ROS indicator carboxy-H_2_DCFDA following the protocol in “Materials” (B).

**Figure 3. (Suppl.):** Original blots and their densitometric analysis were shown. Densitometric analysis of Western blots was carried out using ImageJ software.


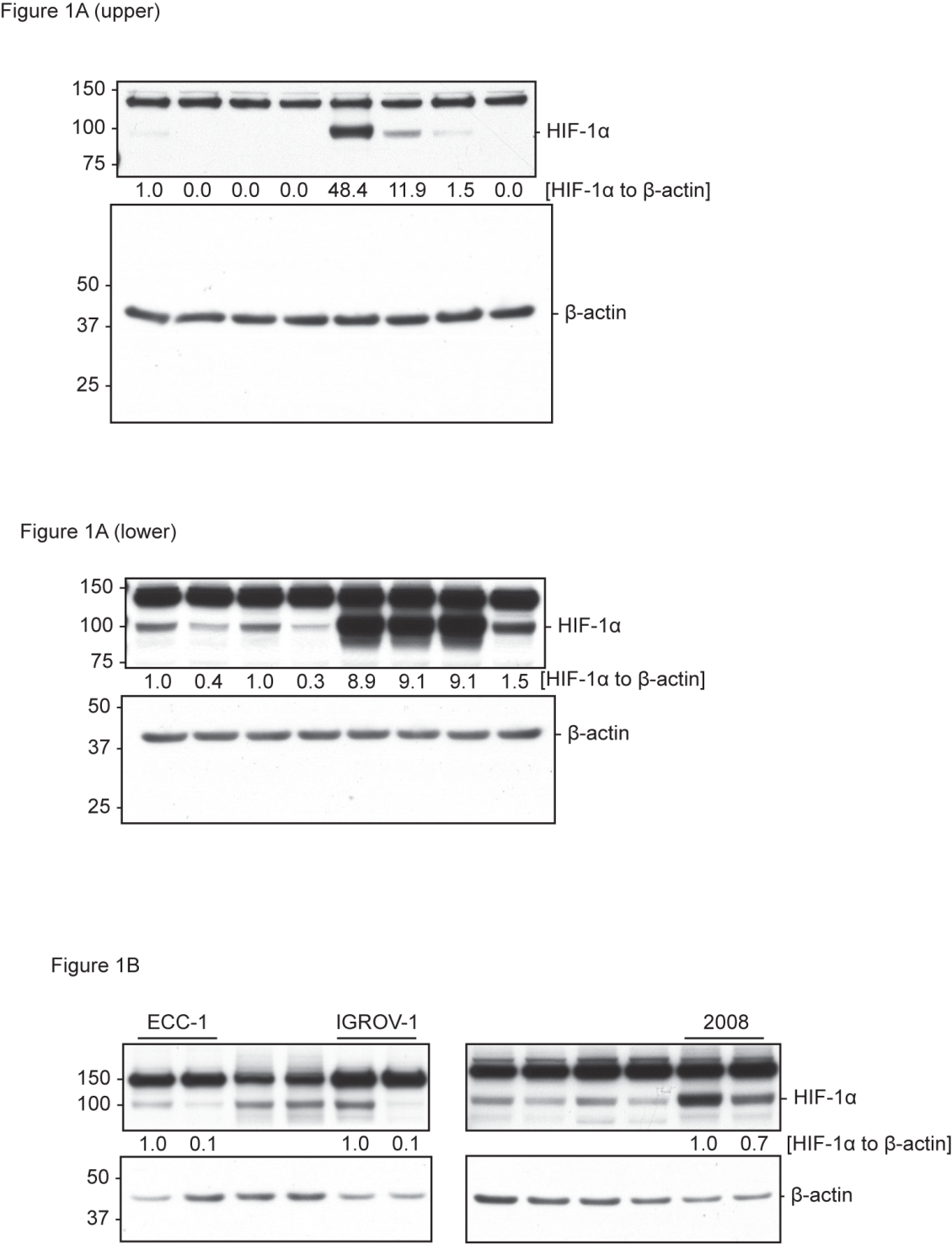


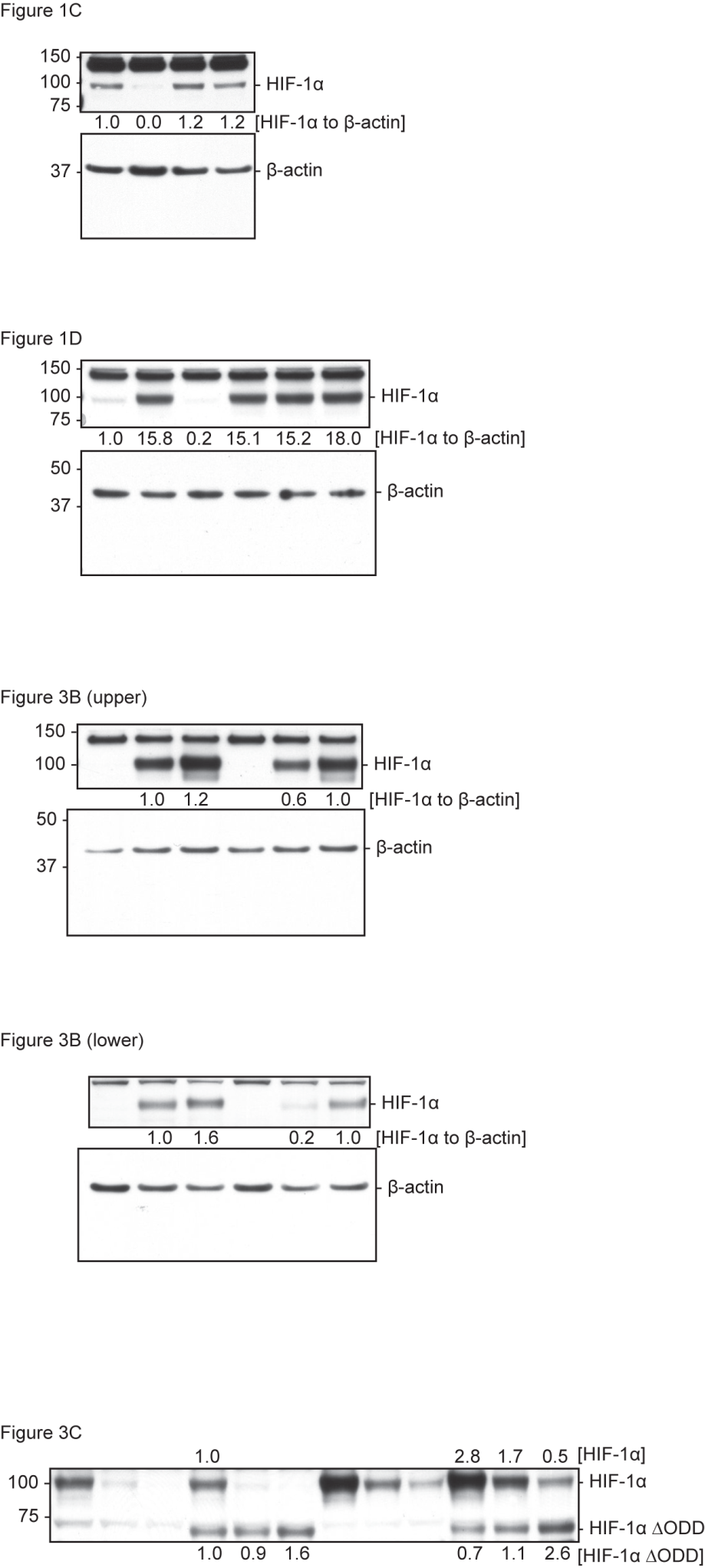


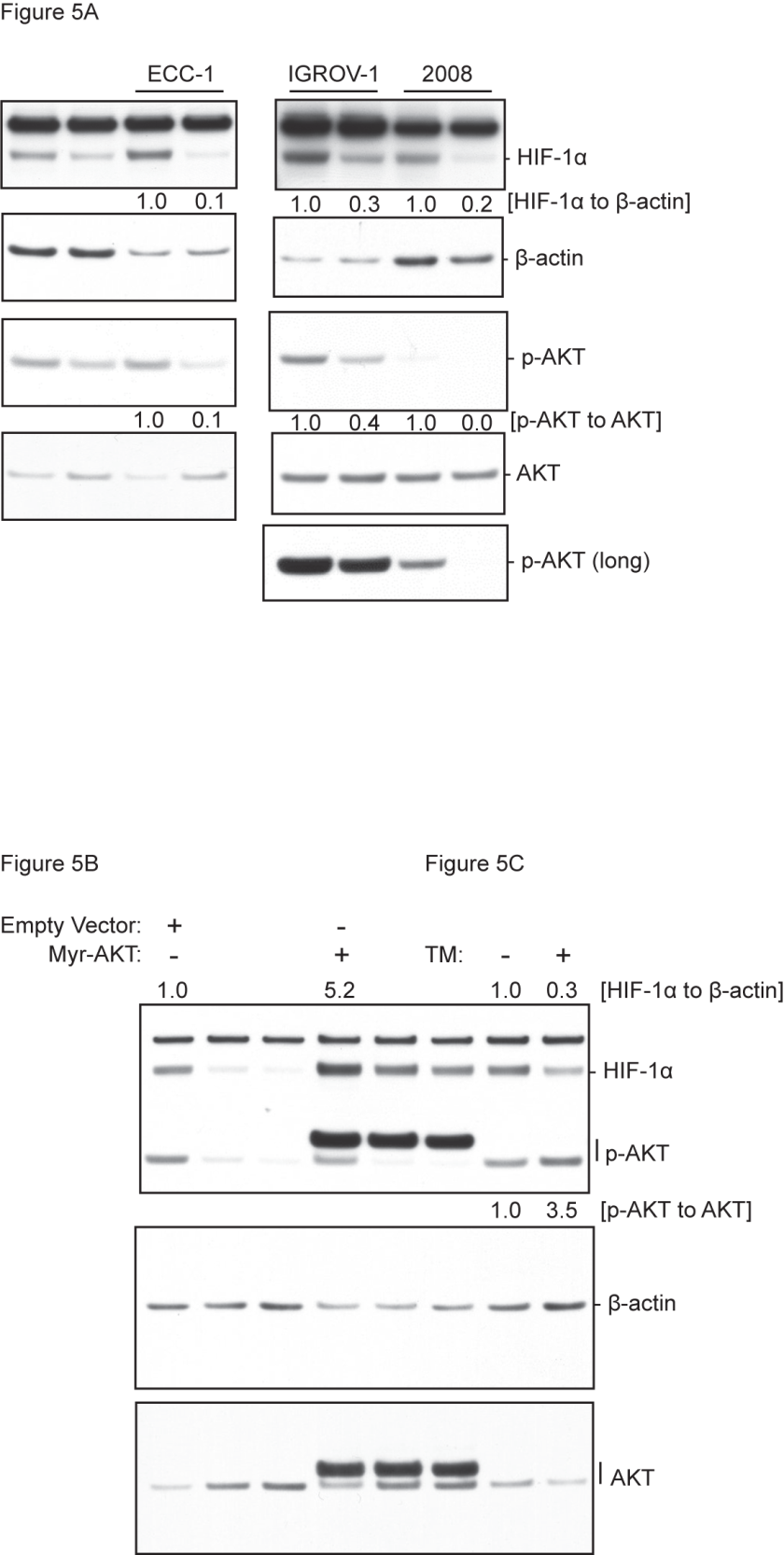

Supplement: Supplementary Information [file srep14296-s1.doc]
